# Functional Bimetal/Carbon Composites Co/Zr@AC for Pesticide Atrazine Removal from Water

**DOI:** 10.3390/molecules28052071

**Published:** 2023-02-22

**Authors:** Danxia Liu, Yongpan Liu, Huijun He, Jie Liu, Xiaolong Yang, Lin Zhang, Yiyan Tang, Hongxiang Zhu

**Affiliations:** 1 College of Environmental Science and Engineering, Guilin University of Technology, Guilin 541004, China; 2 Guangxi Key Laboratory of Environmental Pollution Control Theory and Technology, Guilin 541004, China; 3 Guangxi Collaborative Innovation Center for Water Pollution Control and Water Safety in Karst Area, Guilin 541004, China; 4 Guangxi Modern Industry College of Ecology and Environmental Protection, Guilin 541006, China

**Keywords:** atrazine, cobalt, zirconium, activated carbon, adsorption

## Abstract

Atrazine is a toxic and refractory herbicide that poses threats to human health and the ecological environment. In order to efficiently remove atrazine from water, a novel material, Co/Zr@AC, was developed. This novel material is prepared by loading two metal elements, cobalt and zirconium, onto activated carbon (AC) through solution impregnation and high-temperature calcination. The morphology and structure of the modified material were characterized, and its ability to remove atrazine was evaluated. The results showed that Co/Zr@AC had a large specific surface area and formed new adsorption functional groups when the mass fraction ratio of Co^2+^:Zr^4+^ in the impregnating solution was 1:2, the immersion time was 5.0 h, the calcination temperature was 500 °C, and the calcination time was 4.0 h. During the adsorption experiment on 10 mg/L atrazine, the maximum adsorption capacity of Co/Zr@AC was shown to be 112.75 mg/g and the maximum removal rate was shown to be 97.5% after 90 min of the reaction at a solution pH of 4.0, temperature of 25 °C, and Co/Zr@AC concentration of 60.0 mg/L. In the kinetic study, the adsorption followed the pseudo-second-order kinetic model (R^2^ = 0.999). The fitting effects of Langmuir and Freundlich isotherms were excellent, indicating that the process of Co/Zr@AC adsorbing atrazine also conformed to two isotherm models, so the adsorption of atrazine by Co/Zr@AC had multiple effects including chemical adsorption, mono-molecular layer adsorption, and multi-molecular layer adsorption. After five experimental cycles, the atrazine removal rate was 93.9%, indicating that Co/Zr@AC is stable in water and is an excellent novel material that can be used repeatedly.

## 1. Introduction

Pesticides are becoming increasingly necessary for meeting the demands of agricultural production, but their widespread use pollutes aquatic and terrestrial environments, threatening human health and aquatic life. Atrazine (2-chloro-4-ethylamino-6-isopropylamino-s-triazine) is a common herbicide that is highly effective in controlling broadleaf and grassy weeds [1,2]. Posing a serious threat to the human endocrine and immune systems, atrazine is classified as an endocrine disruptor, and long-term exposure is known to increase cancer risks [3,4]. Atrazine residues are frequently detected in soil, groundwater, and surface water because of the compound’s high mobility and long half-life [5]. Atrazine has been banned in a number of European countries since 1991, but it remains widely used in the United States as well as several developing countries due to its high efficiency and low cost [6]. Recently, atrazine has been used at an annual rate of 70,000–90,000 tons worldwide [7,8].

To mitigate its harmful effects on the ecosystem, several methods have been developed for the removal of atrazine from water, such as photocatalysis [9], adsorption [10], advanced oxidation [11], biodegradation [12], and electrochemistry [13]. Adsorption is a particularly promising method, providing cheap and effective environmental protection. Adsorption methods mainly vary based on the types of adsorbents. Activated carbon, biochar, clay, metal-organic frameworks, and carbon nanotubes [14,15] are commonly used as adsorbents in water treatment due to their high specific surface area, thermal stability, and good regenerative properties [16,17]. Hernandes et al. [18] achieved a 77% atrazine removal rate in 4.0 h using biochar prepared from sawdust, and Muthusaravanan et al. [19] produced graphene oxide nanosheets with an adsorption capacity for atrazine of 138.19 mg/g in water at 45 °C.

Adding new functional groups to adsorbents through surface modification can improve their adsorption capacities, and this technique has become a common practical method in materials science [20]. As one of the most common adsorbents, the removal of pollutants from water by modified activated carbon has been widely studied. For example, Shu et al. [21] prepared Cu@AC using copper-nitrate-modified activated carbon and investigated its adsorption effect on methylene blue (MB); Cu@AC almost entirely removed 400 mg/L of MB from wastewater, with a maximum adsorption capacity of 373 mg/g—1.6 times higher than that of the original activated carbon. Mohammadi et al. [22] prepared a new material (named MCAC) by loading cobalt onto activated carbon, achieving a phenol removal rate of over 90%, as well as good removal rates of other metals, such as chromium, lead, nickel, cadmium, and arsenic. The MCAC was highly reusable, with the regenerated MCAC maintaining an 80% phenol removal efficiency after five cycles of repeated use.

Single metal-loaded materials may exhibit unstable performance and unsatisfactory pollutant removal rates. To address this, a number of studies have investigated the use of bimetals to enhance the performance of activated carbon, reporting greatly improved performance stability and removal performance of pollutants. For instance, Li et al. [23] found that the loading of a Cu–Ni bimetallic oxidation active fraction on activated carbon improved its quinoline removal efficiency by 27% compared with that of activated carbon alone. Liu et al. [24] prepared a novel Fenton catalyst, Fe_3_O_4_-CeO_2_/AC, using the impregnation–precipitation method; imparting Fe_3_O_4_ and CeO_2_ improved the adsorption and conductivity properties of the activated carbon, thereby increasing the catalytic activity of the orange-yellow organic fuel.

In view of the pollution that atrazine creates and the harm it causes to the global ecological environment, how to effectively remove atrazine in the environment is the current research focus, so it is particularly important to develop useful and effective removal materials and technologies. Recent studies have found little evidence of improved performance in zirconium-loaded materials, highlighting a need for further investigation [25]. To the best of our knowledge, there are fewer studies on bimetal-load materials which are loaded by zirconium and cobalt, and there is a paucity of data on the adsorption of atrazine by the bimetal-load active carbon. This study proposes a new bimetal-activated carbon, Co/Zr@AC, prepared by co-loading cobalt and zirconium ions onto activated carbon by solution impregnation and high-temperature calcination to achieve a higher removal rate of atrazine. We investigated the influence of the preparation factors on the material’s properties, and characterized the optimal atrazine removal conditions, its reuse properties, and its removal mechanism. This study is of great significance because it provides insights into the understanding of the use of bimetal-load active carbon in the efficient remediation of herbicide-contaminated water, and also provides a comprehensive reference for other studies.

## 2. Results and Discussions

### 2.1. Effect of Different Preparation Conditions on Material Properties

**Figure 1:**
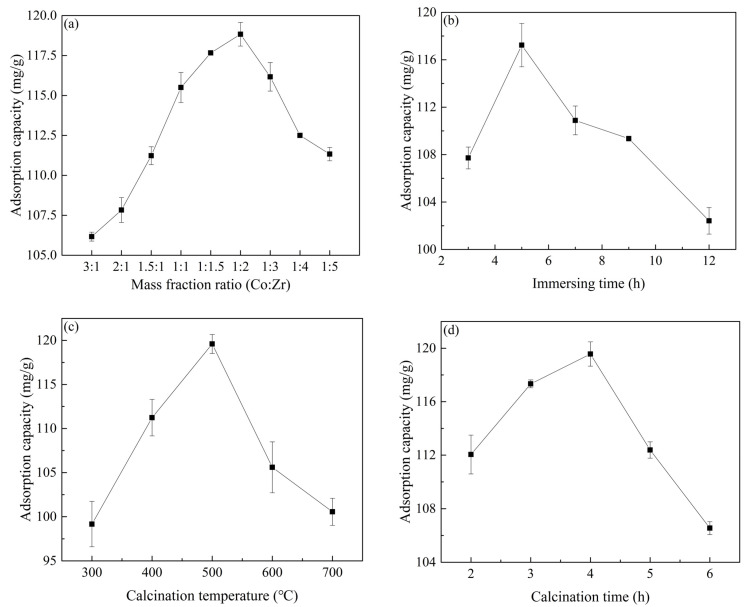
The influence of preparation conditions on material properties: (**a**) mass fraction ratio impregnation solution, (**b**) immersing time, (**c**) calcination temperature, (**d**) calcination time (atrazine concentration 10 mg/L, material dosage 60 mg/L, reaction time 180 min, reaction temperature 25 °C, and shaking speed 120 rpm).

As shown in Figure 1a, the bimetallic mass ratio in the impregnating solution directly affected the performance of Co/Zr@AC. When the Co^2+^/Zr^4+^ ratio was 3:1, the adsorption capacity of Co/Zr@AC for atrazine was the lowest (106.17 mg/g). As the proportion of Zr^4+^ gradually increased, the adsorption capacity of Co/Zr@AC increased, peaking at 118.83 mg/g at a Co^2+^:Zr^4+^ ratio of 1:2. However, further increases in the Zr^4+^ content resulted in a gradual reduction in the adsorption capacity. Compared with a previous study [26], the adsorption capacity of Co/Zr@AC for atrazine was higher than that of Co-ion-loaded AC (92.95 mg/g), Zr-ion-loaded AC (93.37 mg/g), and simple AC (75.66 mg/g). This indicates that there was a synergistic effect between the Co and Zr ions, with the addition of Zr ions enhancing the adsorption effect of the material. The reduced adsorption capacity when the Zr^4+^ content was high could have been a result of the large Zr-containing active fraction covering part of the Co-containing active fraction, causing a reduction in the surface area of the material’s active sites. Thus, an impregnating solution containing the optimal Co^2+^:Zr^4+^ mass fraction ratio of 1:2 was used for all subsequent Co/Zr@AC preparations.

Figure 1b shows the relationship between the immersion time and material adsorption capacity. When the immersion time increased from 3.0 h to 5.0 h, the adsorption capacity of Co/Zr@AC for atrazine increased, reaching a maximum of 117.23 mg/g at 5.0 h. With a longer immersion time, the loading of cobalt and zirconium ions on the activated carbon gradually increased, along with the active fraction, resulting in improved material adsorption. However, due to the dynamic equilibrium relationship between adsorption and desorption and an impregnation time that is too long, metal ions already loaded on activated carbon under the action of agitation may partially desorb, which decreases the amount of metal ions on the activated carbon [27], leading to the decrease in the adsorption capacity of Co/Zr@AC.

Figure 1c shows that the adsorption capacity of Co/Zr@AC for atrazine increased from 99.17 mg/g to 119.6 mg/g as the calcination temperature increased from 300 °C to 500 °C. Further increases in the temperature resulted in a decreased adsorption capacity. When calcined at a lower temperature, the metal ions loaded on the surface of the material were not yet able to fully form stable functional groups, and the content of the active components was low. However, an excessively high calcination temperature may cause the sintering of Co/Zr@AC and the agglomeration of the active components on the surface, reducing the adsorption performance of the material [28,29]. Thus, the optimal calcination temperature of 500 °C was used in the preparation of all subsequent materials.

Figure 1d shows the effect of the calcination time during material preparation on the adsorption capacity of Co/Zr@AC. The adsorption capacity of Co/Zr@AC for atrazine increased with an increase in the calcination time, reaching a peak of 119.57 mg/g at 4.0 h. When the calcination time increased to 6.0 h, the adsorption capacity of Co/Zr@AC for atrazine decreased to 106.55 mg/g. When the calcination time was short, the metal ions could not completely generate functional groups on the surface of the activated carbon, and the active component content was low. With an increase in the calcination time, the adsorption performance of Co/Zr@AC improved, but an excessively long calcination time would cause the material to agglomerate with continual increases in grain size, resulting in a decrease in the specific surface area of Co/Zr@AC. This was confirmed in a subsequent characterization by SEM and FTIR. Therefore, the optimal calcination time of 4.0 h was used in the preparation of all subsequent materials.

In summary, the optimal preparation conditions for Co/Zr@AC included an impregnating solution with a Co^2+^:Zr^4+^ mass fraction ratio of 1:2, an immersion time of 5.0 h, a calcination temperature of 500 °C, and a calcination time of 4.0 h. Unless otherwise specified, the preparation of all subsequent Co/Zr@AC samples was conducted under these conditions.

### 2.2. Characterization

**Figure 2:**
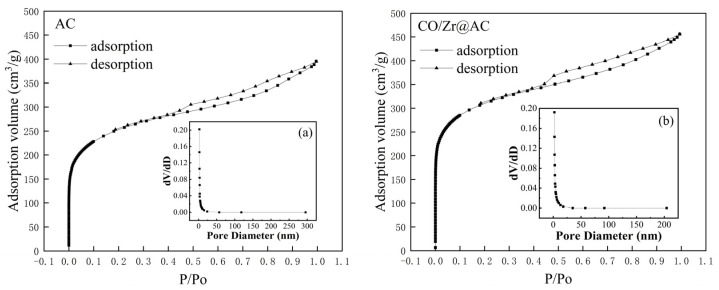
N_2_ absorption–desorption curves and pore size distribution of AC (**a**) and Co/Zr@AC (**b**).

**Figure 3:**
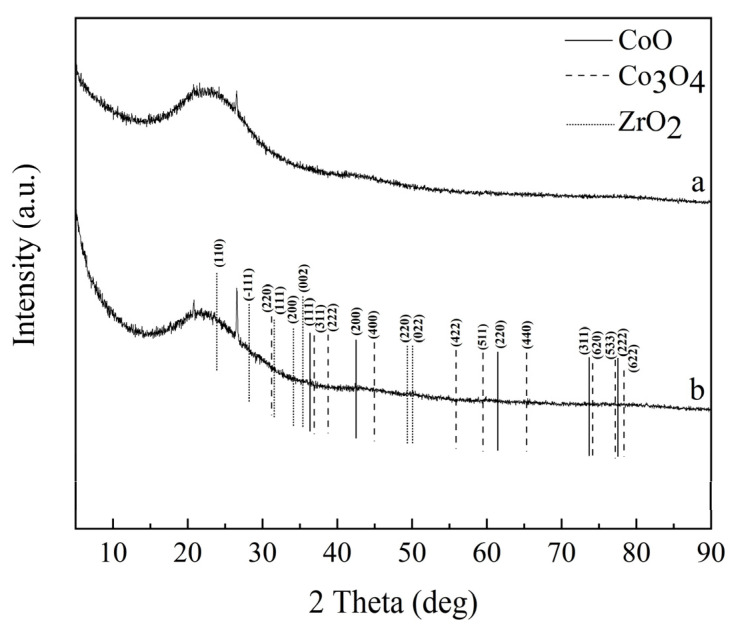
XRD patterns: (**a**) AC, (**b**) Co/Zr@AC (preparation conditions of Co/Zr@AC: mass fraction ratio of Co^2+^: Zr^4+^ 1:2, immersing time 5.0 h, calcining temperature 500 °C, calcination time 4.0 h).

**Figure 4:**
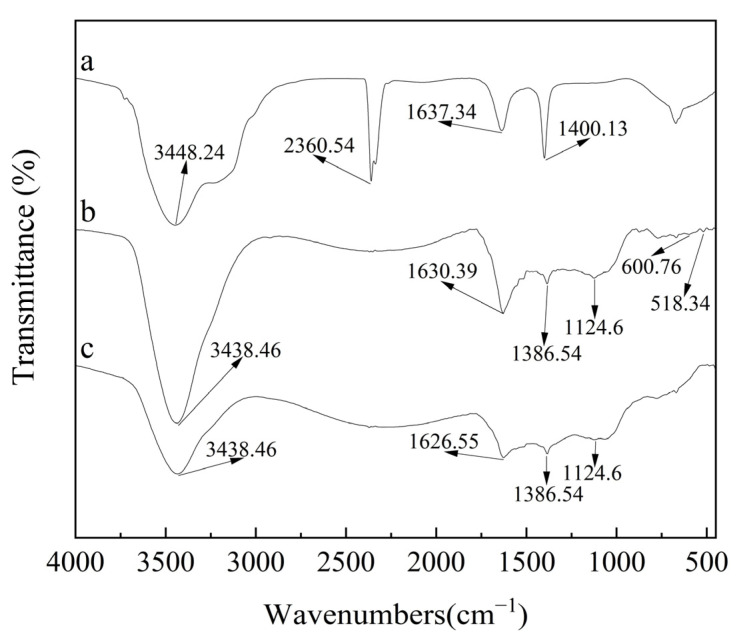
FTIR image: (**a**) AC, (**b**) Co/Zr@AC before adsorption, (**c**) Co/Zr@AC after adsorption (preparation conditions of Co/Zr@AC: mass fraction ratio of Co^2+^:Zr^4+^ 1:2, immersing time 5.0 h, calcining temperature 500 °C, calcination time 4.0 h).

Table 1 shows the results of the specific surface and pore size analyses of the pretreated activated carbon and Co/Zr@AC at optimal conditions. The specific surface area, total pore volume of adsorption, and total pore volume of the micropores of Co/Zr@AC were greater than the those of the pretreated form, indicating the improved adsorption of the activated carbon after bimetallic modification. The nitrogen adsorption–desorption curves of activated carbon and Co/Zr@AC (Figure 2) showed that the highest adsorption/desorption capacity of activated carbon was 395.2 cm^3^/g, whereas the highest adsorption/desorption capacity of Co/@AC was 455.941 cm^3^/g, indicating that the adsorption performance of Co/Zr@AC was improved by impregnation and high-temperature calcination in a certain experimental range. High-temperature calcination may burn the unburned material in the original AC and also lead to the collapse and reorganization of AC pores [22]. At the same time, the loaded metal ions can form new functional groups on the surface of AC (as seen in Figure 3 and Figure 4), increasing the specific surface area of the prepared material. Table 1 illustrates the pore size distribution, which showed that the Co/Zr@AC had a high pore volume around 2.737 nm, indicating that the prepared material was mainly mesoporous (2.0–50.0 nm).

**Figure 5:**
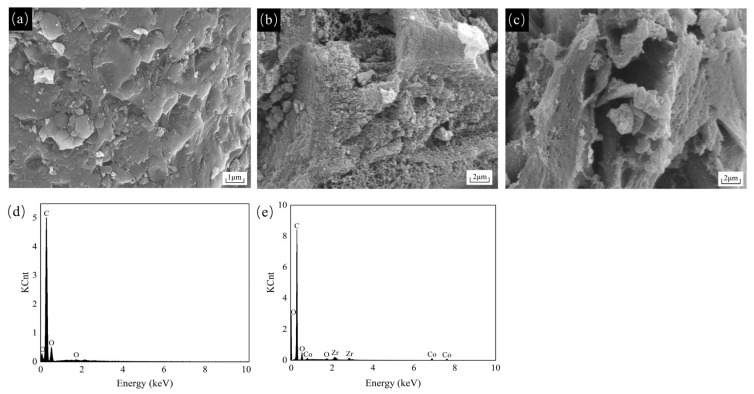
Material characterization: (**a**) SEM image of AC, (**b**) SEM image of Co/Zr@AC calcined for 4.0 h, (**c**) SEM image of Co/Zr@AC calcined for 6.0 h, (**d**) EDS image of AC, (**e**) EDS image of Co/Zr@AC calcined for 4.0 h.

Figure 3 shows the XRD patterns of the activated carbon and Co/Zr@AC. The original activated carbon was almost entirely free of other substances, such as cobalt and zirconium, whereas after modification by bimetals, the Co/Zr@AC had three diffraction signals: CoO, Co_3_O_4_, and ZrO_2_. These diffraction peaks are not notable, owing to the low concentration of the impregnating solution, however, based on the FTIR image and EDS image (Figure 4 and Figure 5e), it also can be proven that the CoO, Co_3_O_4_, and ZrO_2_ diffraction peaks exist in Co/Zr@AC. As shown in Figure 3, CoO displayed five characteristic peaks at 36.49°, 42.39°, 61.5°, 73.67°, and 77.53°, characterizing the (111), (200), (220), (311), and (222) index planes, respectively. Co_3_O_4_ displayed ten characteristic peaks at 31.27°, 36.85°, 38.55°, 44.81°, 55.66°, 59.35°, 65.23°, 74.12°, 77.34°, and 78.4°, characterizing the index planes (220), (311), (222), (400), (422), (511), (440), (620), (533), and (622), respectively [30]. ZrO_2_ had seven characteristic peaks at 24.05°, 28.17°, 31.47°, 34.16°, 35.31°, 49.27°, and 50.12° in the (110), (−111), (111), (200), (002), (220), and (022) index planes, respectively [31]. This indicates that after impregnation and high-temperature calcination, the Co and Zr attached to the activated carbon surface, generating active metal oxide components.

The surface functional groups of AC and Co/Zr@AC were examined by FTIR spectroscopy, and the results are shown in Figure 4. The peaks at 3448.24 cm^−1^ and 3438.46 cm^−1^ correspond to the H-O-H stretching vibrations of adsorbed water [32], the peaks at 2360.54 cm^−1^ were caused by C=O stretching vibrations [33], the peaks at 1637.34 cm^−1^, 1630.39 cm^−1^, and 1626.55 cm^−1^ were caused by C=C stretching vibrations [34], the peaks at 1400.13 cm^−1^ and 1386.54 cm^−1^ are attributed to C-OH [35], and the peak at 1124.6 cm^−1^ is the asymmetric vibration of C-O-C [36]. The C=O vibration peak at 2360.54 cm^−1^ was not present for Co/Zr@AC, whereas a new C-O-C vibration peak was generated at 1124.6 cm^−1^. Moreover, two new characteristic peaks at 600.76 cm^−1^ and 518.34 cm^−1^ appeared on the surface of Co/Zr@AC, the former corresponding to the Co_3_O_4_ spinel structure [30], and the latter corresponding to the absorption peak of Zr-O [37], indicating the presence of new functional groups on the cobalt–zirconium-loaded activated carbon.

Figure 4c shows the FTIR spectra of Co/Zr@AC after adsorption. Compared with the unreacted material, the characteristic peaks of Co/Zr@AC after adsorption are not obvious at 600.76 cm^−1^ and 518.34 cm^−1^. The reason may be that the atrazine adsorbed on the surface of Co/Zr@AC could have masked the group, indicating that the newly generated functional groups were involved in the adsorption process [26].

The SEM images of the activated carbon and Co/Zr@AC are shown in Figure 5. The surface of the original activated carbon was smooth, with very few fine particles attached to it after acid–base pretreatment (Figure 5a), whereas the surface of Co/Zr@AC was rough, and several fine particles were generated (Figure 5b,c). Different particles were produced on the adsorbent surface at different calcination times. The surface of Co/Zr@AC displayed a rougher structure and had a greater amount of active components after 4.0 h of calcination (Figure 5b) than after 6.0 h (Figure 5c), which implies that adsorption was greater after 4.0 h. This also indicates that an excessively long calcination time causes the sintering of the material and the agglomeration of the active components, resulting in a decrease in the specific surface area of Co/Zr@AC, as is consistent with the results shown in Figure 1d. Figure 5e displays the EDS plot of Co/Zr@AC after 4.0 h of calcination, showing that Co and Zr were successfully loaded onto the activated carbon.

### 2.3. Effect of Different Adsorption Conditions on Atrazine Removal

**Figure 6:**
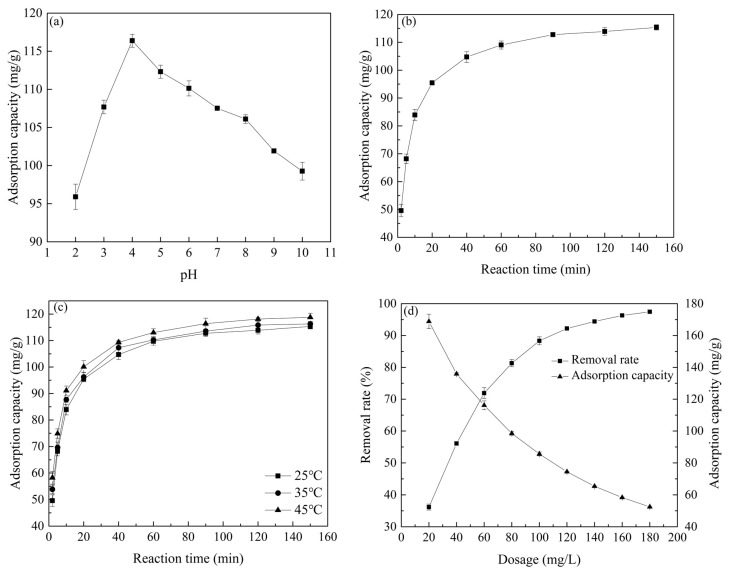
The effect of the reaction conditions on the removal of atrazine: (**a**) solution pH, (**b**) reaction time, (**c**) reaction temperature, (**d**) material dosage.

To systematically evaluate the atrazine removal efficiency of Co/Zr@AC, the effects of four factors, which are solution pH, adsorption time, adsorption temperature, and adsorbent concentration, were studied (see Figure 6). 

Solution pH is one of the key factors affecting the removal of atrazine by adsorbents [38]. As shown in Figure 6a, the adsorption capacity of Co/Zr@AC for atrazine increased rapidly when the pH increased from 2.0 to 4.0, reaching a peak at a pH of 4.0. When the pH was further increased, the adsorption capacity of Co/Zr@AC gradually decreased to 99.27 mg/g at pH 10.0. Related studies have shown that atrazine is negatively charged in solution [39]; this indicates that, in moderately or weakly acidic conditions (e.g., 4.0 ≤ pH < 7.0), dissolved H^+^ ions adsorb on the surface of the material, forming positively charged functional groups that readily adsorb negatively charged atrazine molecules. However, in a strongly acidic solution (e.g., pH = 2.0–3.0), a large amount of free H^+^ neutralizes the negatively charged atrazine, which causes the atrazine’s negative charge to decrease or even become positive. As Co/Zr@AC is also positively charged under these conditions due to the adsorption of H^+^, electrostatic repulsion occurs between atrazine and the adsorbent, weakening its adsorption ability. In alkaline solutions, the large number of OH^-^ ions causes the functional groups on the adsorbent’s surface to become negatively charged, resulting in repulsion between Co/Zr@AC and atrazine, which reduces the material’s adsorption capacity. This is consistent with the results of Wei et al., which indicated that atrazine is more efficiently removed under acidic conditions [40,41,42].

Figure 6b shows that the adsorption capacity of Co/Zr@AC for atrazine continued to increase with increasing adsorption time and reached the adsorption equilibrium of 112.75 mg/g at 90 min. The rapid increase in the adsorption capacity of the material during the first 20 min indicates that the removal of atrazine by Co/Zr@AC is a rapid adsorption process and that the most readily available adsorption sites on the surface of the material were rapidly utilized. As we proceeded, adsorption on the surface of Co/Zr@AC increased.

Figure 6c shows the relationship between the adsorption temperature and the adsorption capacity of Co/Zr@AC. There was a positive correlation between adsorption capacity and temperature at the same reaction time. This suggests that higher temperatures promote the molecular movement of atrazine in solution, increasing both the chance of contact with Co/Zr@AC and the energy exchanged upon contact, thereby accelerating the adsorption process [43].

Figure 6d shows that the atrazine removal rate increased gradually with an increase in the Co/Zr@AC concentration. As the concentration increased from 20 mg/L to 180 mg/L, the removal rate increased from 36.16% to 97.45%. This was because of the greater adsorbent surface area and the larger number of adsorption sites present at higher concentrations. Conversely, an increase in the concentration caused a reduction in Co/Zr@AC’s adsorption capacity. This is attributed to the fact that all adsorbents were involved in the adsorption when a small amount of adsorbent was present in the solution, but at high concentrations, not all adsorption sites could participate effectively in the adsorption process, which led to the decrease in the material’s adsorption capacity.

In summary, at a solution pH of 4.0 and a reaction temperature of 25 °C, the optimal adsorption capacity of 60 mg/L Co/Zr@AC for atrazine is 112.75 mg/g after 90 min of reaction.

### 2.4. Adsorption Kinetics and Adsorption Isotherms

**Figure 7:**
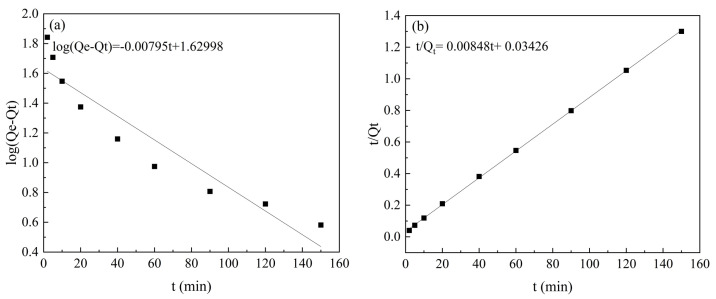
Co/Zr@AC adsorption kinetics of atrazine: (**a**) pseudo-first-order kinetic model, (**b**) pseudo second-order kinetic model (Co/Zr@AC dosage 60 mg/L, atrazine concentration 10 mg/L, solution pH value 4.0, reaction temperature 25 °C).

The results of the adsorption kinetic modelling are shown in Figure 7 and Table 2. The correlation coefficient (R^2^) of the pseudo-second-order kinetic model was 0.999; this was significantly higher than that of the pseudo-first-order kinetic model (R^2^ = 0.885). In addition, the theoretical adsorption capacity (Q_cal_ = 117.925 mg/g) calculated by the pseudo-second-order kinetic model was closer to the experimental value (Q_exp_ = 112.75 mg/g), indicating that the pseudo-second-order kinetic model is more suitable for simulating this adsorption process, and that Co/Zr@AC mainly adsorbs atrazine through chemisorption.

**Figure 8:**
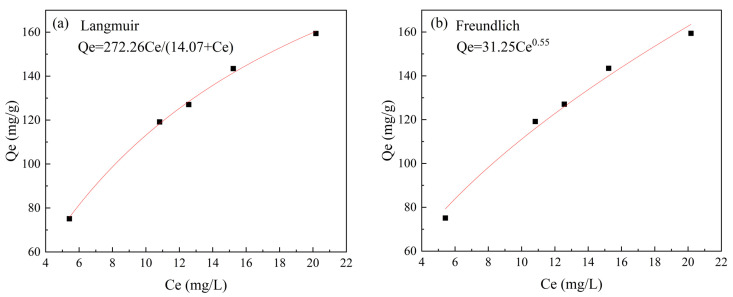
The adsorption isotherms of Co/Zr@AC for atrazine: (**a**) Langmuir isotherm, (**b**) Freundlichisotherm (Co/Zr@AC dosage 60 mg/L, atrazine concentration 10 mg/L, solution pH value 4.0, reaction time 90 min, reaction temperature 25 °C).

The results of the Langmuir and Freundlich isotherm adsorption isotherm fits are shown in Figure 8 and Table 3. The fit of the Langmuir isotherm adsorption model (R^2^ = 0.997) was better than that of the Freundlich isotherm adsorption model (R^2^ = 0.981), indicating that the material adsorbs atrazine primarily as a single molecular layer [44].

**Figure 9:**
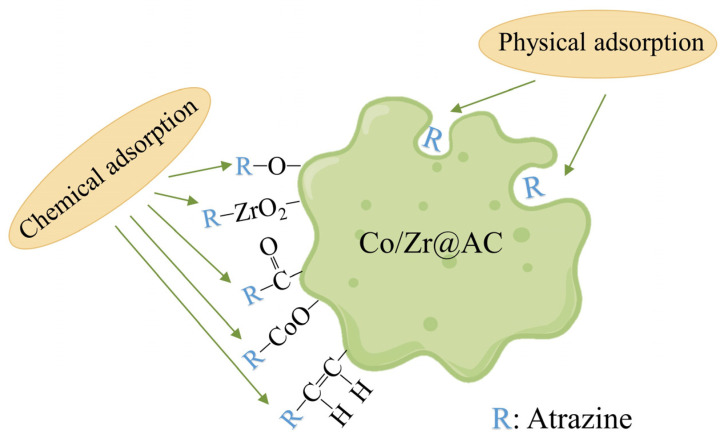
Schematic diagram of the mechanism of Co/Zr@AC’s adsorption of atrazine.

As mentioned above, the mechanism of the adsorption of atrazine by Co/Zr@AC is shown in Figure 9. Due to the large number of micropores in Co/Zr@AC, it has the physical adsorption properties required for atrazine removal. At the same time, the material contains a large number of functional groups, as shown in Figure 4 and Figure 5, which can remove atrazine through chemical adsorption. It is because of these adsorption properties that the material can efficiently remove atrazine.

Table 4 showed the adsorption capacity of the developed adsorbent with that of the other materials reported in the literature for atrazine removal. The results proved significant differences in maximum adsorption capacity (Q_max_) between these materials. The adsorption capacity of Co/Zr@AC (Q_max_ = 112.75 mg/g) was the highest in Table 4, confirming its highly efficient adsorption of atrazine. These results show that the active sites on the surface of the material obtained by the modified activated carbon were well distributed, thus achieving efficient pollutant adsorption.

### 2.5. Cycle Experiments

**Figure 10:**
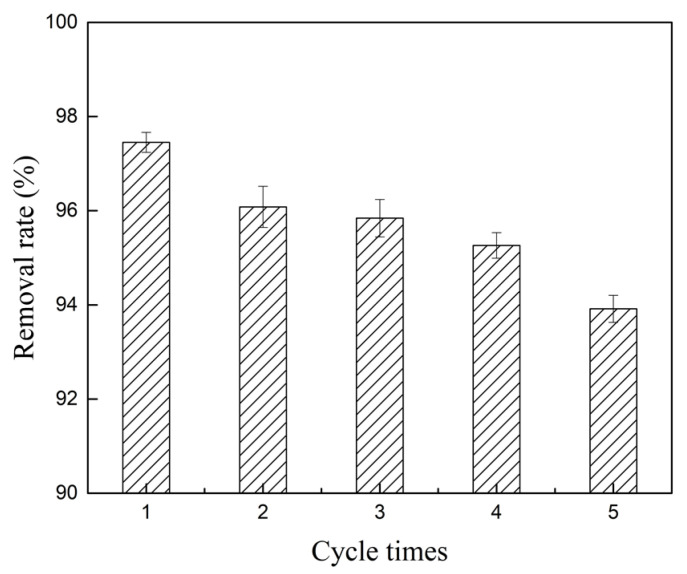
Cycle experiments on Co/Zr@AC (Co/Zr@AC dosage 180 mg/L, atrazine concentration 10 mg/L, solution pH 4.0, reaction time 180 min, reaction temperature 25 °C, and shaking speed 120 rpm).

Service life and reuse rate are important factors in the practical application of materials. Figure 10 shows that after five cycles of experiments, the removal rate was 93.92%, indicating that Co and Zr can be stably loaded on AC by solution impregnation and high-temperature calcination. The prepared Co/Zr@AC showed good removal performance and stability after multiple cycles, indicating that it is a promising recyclable material.

## 3. Materials and Methods

### 3.1. Materials

Activated carbon was purchased from Xilong Scientific Co. (Guangzhou, China). The Brunauer–Emmett–Teller (BET) specific surface area was 881.338 m^2^/g, its total pore volume was 0.611 cm^3^/g, its mean aperture was 2.853 nm, and its point-zero charge pH (pH_zpc_) was 6.3. The activated carbon was pretreated as follows: the powdered activated carbon was screened with a standard 100 mesh sample sieve. The undersized powder was soaked in a 0.5 mol/L NaOH solution for 24.0 h, then washed with Milli-Q water (>18 MΩ cm) until neutral, and dried in an oven at 90 ℃. Subsequently, the treated activated carbon was soaked in a 1.0 mol/L HCl solution for 24.0 h, filtered, washed with Milli-Q water until neutral, and subsequently dried in an oven at 90 ℃. The final product was hydrogen-type activated carbon.

The pesticide atrazine (>97% purity) used in the experiment was procured from Shanghai Yuanye Biological Technology Co., Ltd., China. Unless otherwise stated, the concentration of atrazine used in all experiments was 10 mg/L. A cobalt and zirconium ion stock solution with a mass fraction of 7% was prepared by dissolving cobalt nitrate [Co(NO_3_)_2_•6H_2_O] and zirconium nitrate [Zr(NO_3_)_4_•5H_2_O] in Milli-Q water. All chemicals were analytically pure and did not require further purification, except for methanol and acetonitrile, which were chromatographically pure for the liquid chromatography analysis.

### 3.2. Preparation of Co@AC

The pretreated activated carbon was impregnated with the cobalt and zirconium ion stock solution, the sample was filtered and dried at 90 °C, and the cobalt and zirconium ions were stably loaded on the activated carbon by high-temperature calcination. Finally, the calcined solid was ground into a powder to produce Co/Zr@AC.

Single-factor tests were used to investigate the effects of the impregnating solution’s Co^2+^:Zr^4+^ mass fraction ratio (3:1, 2:1, 1.5:1, 1:1, 1:1.5, 1:2, 1:3, 1:4, and 1:5), immersion time (3.0, 5.0, 7.0, 9.0, and 12.0 h), calcination temperature (300, 400, 500, 600, and 700 °C), and calcination time (2.0, 3.0, 4.0, 5.0, and 6.0 h) on the material’s properties. Each single-factor test assumed specific values for the factors that were not of interest. These included an impregnating solution bimetallic mass fraction ratio of 1:1, an immersion time of 7.0 h, a calcination temperature of 500 °C, and a calcination time of 4.0 h. The performances of the materials were based on their removal effects on 10 mg/L atrazine, and they were characterized and analyzed using high-precision instruments.

### 3.3. Atrazine Removal Experiment

Different concentrations of Co/Zr@AC were added to conical flasks containing 50 mL of atrazine solution (the concentration of atrazine was 10 mg/L) and placed in a constant-temperature water bath shaker at 120 rpm. After 3.0 h, the solution was filtered through a 0.45 μm filter membrane, and the concentration of atrazine in the filtrate was analyzed by liquid chromatography. The results of liquid chromatography were used to calculate the atrazine removal rate and the adsorption capacity of Co/Zr@AC.

The effect of solution pH (2.0, 3.0, 4.0, 5.0, 6.0, 7.0, 8.0, 9.0, and 10.0), reaction time (2, 5, 10, 20, 40, 60, 90, 120, and 150 min), reaction temperature (25, 35, and 45 °C), and Co/Zr@AC concentration (20.0, 40.0, 60.0, 80.0, 100.0, 120.0, 140.0, 160.0, and 180.0 mg/L) on the removal of atrazine were investigated using single-factor tests. In this experiment, the assumed values of the solution pH, reaction time, reaction temperature, and adsorbent concentration were 6.0 h, 180 min, 25 °C, and 60 mg/L, respectively.

### 3.4. Analysis Method

The atrazine concentrations were measured at 236 nm with a high-performance liquid chromatograph (HPLC, 1260 Infinity, Agilent Technologies) equipped with a UV detector and an Agilent ZORBAX SB C18 (5.00 μm × 4.60 mm × 250 mm) reverse phase column. HPLC was operated according to the mobile phases of water, acetonitrile, and methanol (30:10:60,) at 1.00 mL/min. Twenty microliters of the sample was injected and detected at a column temperature of 40 °C and a retention time of 8.5 min.

The formulae for the adsorption capacity (Equation (1)) and removal rate (Equation (2)) are as follows:

(1) $$Q_{t}=\frac{V\;\times \;\left(C_{0}{\;-\;C}_{t}\right)}{m},$$

(2) $$\eta =\frac{C_{0}{\;-\;C}_{t}}{C_{0}}\times 100\%,$$

where Q_t_ (mg/g) is the adsorption capacity at time t, V (L) is the volume of the atrazine solution, C_0_ (mg/L) is the initial atrazine concentration, C_t_ (mg/L) is the remaining atrazine concentration at time t, m (g) is the adsorbent mass, and η (%) is the atrazine removal rate.

Primary and secondary kinetic models were proposed to describe the adsorption kinetics of Co/Zr@AC on atrazine, and the Langmuir and Freundlich isothermal adsorption models were used to analyze the adsorption equilibrium relationship [55]. The formulae of the pseudo-first-order kinetic model (Equation (3)), pseudo-second-order kinetic model (Equation (4)), Langmuir isothermal adsorption model (Equation (5)), and Freundlich isothermal adsorption model (Equation (6)) are as follows:

(3) $$\log\left(Q_{e}{\;-\;Q}_{t}\right){=\mathrm{logQ}}_{e}-\frac{k_{1}}{2.303}t,$$

(4) $$\frac{t}{Q_{t}}=\frac{1}{k_{2}Q_{e}^{2}}+\frac{1}{Q_{e}}t,$$

(5) $$Q_{e}=\frac{Q_{\max}C_{e}}{K_{L}+C_{e}},$$

(6) $$Q_{e}=K_{f}C_{e}^{\frac{1}{n}},$$

where Q_e_ (mg/g) is the adsorption amount at equilibrium, Q_t_ (mg/g) is the adsorption amount at time t, k_1_ is the pseudo-first-order adsorption rate constant, k_2_ is the pseudo-second-order adsorption rate constant, C_e_ (mg/L) is the concentration of atrazine at adsorption equilibrium, Q_max_ (mg/g) is the saturated adsorption amount, K_L_ (L/mg) is the affinity constant of the Langmuir equation, and K_f_ ((mg/g)/(mg/L)^n^) and n (g/L) are constants of the Freundlich equation.

### 3.5. Characterization

The specific surface area, pore size, and nitrogen isothermal adsorption and desorption curves of Co/Zr@AC were determined using a specific surface and pore size analyzer (BET, JW-BK200C, Beijing, China). The surface morphology and microzone composition of the materials were analyzed by scanning electron microscopy (SEM; ∑IGMA, Zeiss, Germany) and energy dispersive spectroscopy (EDS), and the samples were measured by dispersing the sample powder onto the conductive gel and spraying gold on the surface for 30 s. Material phase analysis was performed by X-ray diffraction (XRD; X’Pert3 Powder, PANalytical B.V. Netherlands). Finally, Fourier-transform infrared absorption spectrometry (FTIR; NICOLET iS10, Thermo Fisher, USA) was used to determine the surface functional groups of the samples. The dried samples were mixed with KBr and ground into powder at a mass ratio of 1:100 and measured in the range 450–4000 cm^−^^1^.

### 3.6. Desorption and Cycle Experiments

Once the removal experiment was completed, the obtained Co/Zr@AC was filtered through a 0.45 μm organic system filter membrane, rinsed five times with a mixture of pure methanol and ultrapure water (1:1, *v*/*v*), then rinsed with ultrapure water before being dried at 90 °C to a constant weight. This cycle was repeated to test the reusability of Co/Zr@AC according to the test method described in Section 2.3.

Three parallel samples were set up for all tests, and the average value was used as the experimental result.

## 4. Conclusions 

In this study, a new high-performance material, Co/Zr@AC, was successfully prepared by loading bimetallic cobalt and zirconium onto activated carbon via solution impregnation and high-temperature calcination. Under optimal preparation conditions (an impregnation solution of Co^2+^:Zr^4+^ with the mass fraction ratio of 1:2, immersion time of 5.0 h, calcination temperature of 500 °C, and calcination time of 4.0 h), the prepared Co/Zr@AC had a large specific surface area (1078 m^2^/g) and a high total pore volume (0.705 cm^3^/g), and new functional groups were generated. The adsorption capacity peaked at 112.75 mg/g after 90 min of the reaction at a pH of 4.0 and a temperature of 25 °C. The mechanism study revealed that the pseudo-second-order kinetic model and the Langmuir isothermal adsorption model are more suitable for simulating this adsorption process than the pseudo-first-order kinetic model and the Freundlich isotherm model are, indicating that the adsorption involves both chemisorption and monolayer adsorption. Cyclic experiments showed that Co/Zr@AC is a promising and reusable material. Overall, this study has shown that Co/Zr@AC can be used as an efficient material for the removal of organic herbicide from water. At the same time, on the basis of research in this field, it is possible to consider finding suitable microorganisms and using joint material–microbial technology to prepare novel materials to improve the removal of organic herbicide. In order to reduce the leaching of metal ions from the adsorbent during usage, the method of preparing the materials needs to be further improved. In addition, better desorption methods need to be investigated in order to improve the cycle times of materials. Moreover, the post-treatment of used adsorbents should also be the focus of this field.

## Figures and Tables

**Table 1:** BET surface area parameters of AC and Co/Zr@AC.

	Specific Surface Area (m^2^/g)	Total Pore Volume (cm^3^/g)	Micropore Volume (nm)	Pore Size (nm)
AC	881	0.611	0.371	2.853
Co/Zr@AC	1079	0.705	0.458	2.737

**Table 2:** The constants of pseudo-first-order kinetic and pseudo-second-order kinetic models for the adsorption of atrazine by Co/Zr@AC.

Pseudo-First-Order Kinetic Model			Pseudo-Second-Order Kinetic Model		
k_1_	Q_cal_(mg/g)	R^2^	k_2_	Q_cal_(mg/g)	R^2^
0.0183	42.656	0.885	0.0021	117.925	0.999

**Table 3:** The Langmuir and Freundlich isotherm constants of Co/Zr@AC for atrazine.

Langmuir Isotherm			Freundlich Isotherm		
Q_max_ (mg/g)	K_L_ (L/mg)	R^2^	K_f_ ((mg/g)/(mg/L)^n^)	n (g/L)	R^2^
272.26	14.07	0.997	31.25	1.82	0.981

**Table 4:** The adsorption capacity of different adsorbents in the literature for the adsorption of atrazine.

Adsorbent	T (K)	C_0_(mg/L)	S_BET_ (m^2^/g)	Q_m_ (mg/g)	Reference
MgO modified fallen leaf biochar	298	2–12	93	22.40	[45]
Commercial organophilic clay	298	10	2.13	2.28	[46]
Chitosan-modified sepiolit	298	2–20	71	17.92	[47]
surfactant modified clay	298	5	/	4.24	[48]
Cellulose nanofiber modified polymeric cryogels	298	250	/	95.76	[49]
Activated carbon from *Hovenia dulcis*	298	30	898	58.00	[50]
Activated carbon with FeCl_3_	298	5–40	431	55.85	[51]
Magnetic carbon nanotubes	298	1–30	96	57.80	[52]
HCl modified corncob biochar	298	1–25	350	19.58	[53]
N-doped biochars	298	5	700	103.60	[54]
Co/Zr@AC	298	10	1079	112.74	This study

## Data Availability

The data presented in this study are available on request from the corresponding author.

## Sample Availability

Samples of the compounds are available from the authors.
